# Loss and regain of SIV control upon CD8+ cell depletion in vivo in SIV-controller macaques is not associated with efficient SIV specific CD8+ T-cells

**DOI:** 10.1186/1742-4690-9-S2-P287

**Published:** 2012-09-13

**Authors:** C Hamimi, T Bruel, N Dereuddre-Bosquet, A Cosma, S Shin, A Corneau, P Versmisse, C Torres, B Delache, S Even, S Guenounou, B Targat, B Malleret, I Karlsson, F Barré-Sinoussi, R Le-Grand, G Pancino, A Saez-Cirion, B Vaslin

**Affiliations:** 1 Institut pasteur, Paris, France; 2 CEA, Division of Immuno-Virology, Institute of Emerging Diseases and Innovative Therapy, Paris, France

## Background

Spontaneous long-term HIV/SIV control in HIV-controller patients and SIV-controller macaques (SIC) is usually associated to protective MHC-class-I alleles and efficient CD8 T-cell responses. However, many HIV-controllers efficiently control HIV-infection despite of non-protective MHC background and weak CD8 T-cell responses, raising the question of real contribution of this response in maintaining viral control. We addressed this question by depleting in vivo CD8+ cells in SIC bearing or not protective MHC and weak CD8 T-cell responses.

## Methods

We studied five SIVmac_251_</sub>-infected cynomolgous macaques which maintained viremia <400 RNA copies/ml during 5 years. We transiently depleted CD8+ cells in vivo in these animals by injection of anti-CD8 mAb. We analysed, in blood and tissues, the viral load evolution, T-cell frequency and activation, SIV-specific T-cell functionality and plasma cytokine levels.

## Results

CD8+ -depletion in blood and tissues lasted between 10-21 days. One SIC kept undetectable viremia during depletion despite carrying infectious and in vitro inducible SIVmac_251_ in CD4 T-cells. Four SIC experienced a viral rebound (3.10^3^ -7.10^4^ RNA copies/ml) but were able to subsequently re-control viremia to baseline levels (17-21 days post-depletion). In two SIC, regain of viral control started despite CD8+ T-cells being still undetectable. In the two other SIC, regain of viral control coincided temporally with the recovery of CD8 T-cells. However, CD8 T-cell recovery was accompanied by relatively weak expansion of SIV-specific CD8 T-cells with no acquisition of effector antiviral functions. Depletion provoked induction of pro-inflammatory cytokines and homeostatic activation/expansion of CD4+ T-cells, which correlated with plasma viremia (p=0.0006).

## Conclusion

Our results suggest that SIV-specific CD8 T-cell responses are likely not the major contributor for the long-term maintainance of SIV-control in this SIC model. Other mechanisms, including weak viral reservoirs and control of activation, may contribute to viral control. Our SIC model will be useful to investigate mechanisms contributing to natural HIV-control in humans.

